# Transport of a Micro Liquid Plug in a Gas-Phase Flow in a Microchannel

**DOI:** 10.3390/mi9090423

**Published:** 2018-08-23

**Authors:** Yutaka Kazoe, Takumi Matsuno, Ippei Yamashiro, Kazuma Mawatari, Takehiko Kitamori

**Affiliations:** Department of Applied Chemistry, Graduate School of Engineering, The University of Tokyo, 7-3-1 Hongo, Bunkyo, Tokyo 113-8656, Japan; kazoe@icl.t.u-tokyo.ac.jp (Y.K.); takumi.matsuno@gmail.com (T.M.); yamashiro@icl.t.u-tokyo.ac.jp (I.Y.); kmawatari@icl.t.u-tokyo.ac.jp (K.M.)

**Keywords:** microfluidics, microchannel, droplet

## Abstract

Micro liquid droplets and plugs in the gas-phase in microchannels have been utilized in microfluidics for chemical analysis and synthesis. While higher velocities of droplets and plugs are expected to enable chemical processing at higher efficiency and higher throughput, we recently reported that there is a limit of the liquid plug velocity owing to splitting caused by unstable wetting to the channel wall. This study expands our experimental work to examine the dynamics of a micro liquid plug in the gas phase in a microchannel. The motion of a single liquid plug, 0.4–58 nL in volume, with precise size control in 39- to 116-m-diameter hydrophobic microchannels was investigated. The maximum velocity of the liquid plug was 1.5 m/s, and increased to 5 m/s with splitting. The plug velocity was 20% of that calculated using the Hagen-Poiseuille equation. It was found that the liquid plug starts splitting when the inertial force exerted by the fluid overcomes the surface tension, i.e., the Weber number (ratio of the inertial force to the surface tension) is higher than 1. The results can be applied in the design of microfluidic devices for various applications that utilize liquid droplets and plugs in the gas phase.

## 1. Introduction

Microfluidics has rapidly developed, leading to practical applications in bioanalysis, medical diagnosis, and chemical synthesis [1,2]. Various chemical operations, such as mixing, separation, and reaction, have been miniaturized in microchannels [3], and can be integrated to achieve fast and highly efficient chemical processing in a small volume (pL to nL).

In order to realize such microchemical systems, multiphase flows in the microchannels have been utilized [3,4]. Gas-liquid segmented droplet/plug flows have several advantages, such as compartmentalization by the gas phase to eliminate contamination and enable a higher flow rate [5,6,7], compared to liquid-liquid droplet/plug flows, which are widely used in microfluidics [4]. By utilizing segmentation by the gas phase and circulating flow in liquid plugs, mixing is significantly enhanced, with a 2- to 3-fold reduction in mixing length, compared with that achieved using micromixers that utilize chaotic flows induced by microstructures [8]. This characteristic has been applied to achieve ultrafast and uniform chemical synthesis of colloidal silica [9], magnetic nanoparticles [10], quantum dots [11], and gold nanoparticles [12]. The circulating flow in a segmented droplet in the gas phase has been applied to enhance heat transfer [13]. By utilizing a gas phase, whose viscosity is 100 times lower than that of a liquid phase, we previously developed a microdroplet collider that exploits spatially and temporally localized kinetic energy in a microspace [14]. A nL liquid plug in the gas phase was accelerated and made to collide with a target liquid plug. Due to the fluid flow generated by the collision, 6000 times faster mixing was achieved compared to that achieved using molecular diffusion.

Based on these previous studies, higher velocities of liquid droplets and plugs in the gas phase in a microchannel are expected to enable chemical processing with higher efficiency and higher throughput. However, we previously investigated the behavior of a single liquid plug in the gas phase in a hydrophobic microchannel and found that the plug velocity is limited by the splitting due to wetting of the plug tail to the channel wall [15]. Most recently, we found that when the channel diameter is 39 μm, the maximum velocity of a 3.4-nL plug is ~5 m/s [16]. Therefore, understanding the behavior of a liquid plug in the gas phase in a microchannel is important in the design of microfluidic devices that utilize gas-liquid segmentation.

The objective of the present study is to examine the dynamics of a nL liquid plug in the gas phase flowing through a microchannel. The size of the liquid plug was precisely controlled with a Laplace valve developed by our group [17]. A single liquid plug was formed in the microchannel by utilizing the channel geometry and surface wettability. The behavior of the single liquid plug was observed. The plug acceleration and splitting are discussed in terms of fluid dynamics.

## 2. Experimental Section

The experimental setup and experimental procedure were similar to those in our previous study [16]. The experimental setup consisted of an inverted microscope combined with 2× and 4× objective lenses and a high-speed complementary metal-oxide semiconductor (CMOS) camera (FASTCAM, Photron Ltd., Tokyo, Japan) with a pixel size of 20 μm. An optical fiber illumination system (PCS-HRX, NPI Co. Ltd., Tokyo, Japan) in addition to normal microscope illumination was used to enhance image brightness. The frame rate range used to capture the images was 13,333 to 25,000 Hz. To drive fluids in the microchannel, a high-pressure control system that uses compressed air, which was developed by our group [18], was used. In this system, the compressed air generated by a compressor is stored in a tank with a solenoid valve. When the solenoid valve is opened, the pressurized air from the tank is applied to a sample in a reservoir connected to a microchip.

**Figure 1:**
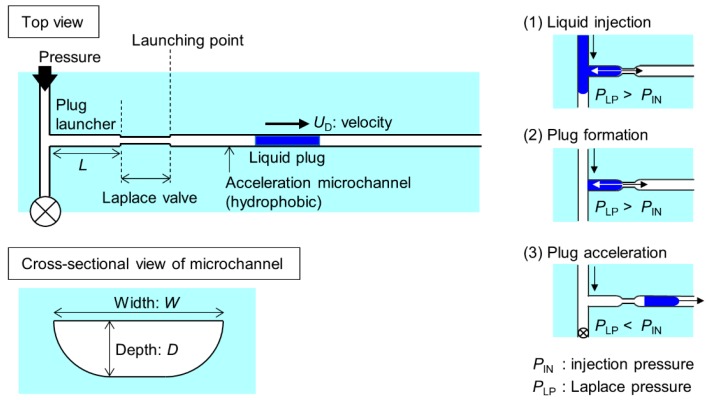
Schematic diagrams of fluidic operations in the experiments utilizing a glass microchannel whose surface was modified to be hydrophobic.

As illustrated in Figure 1, the microchip consisted of a plug launcher, a Laplace valve, which is narrower than other channels, and an acceleration microchannel. The channel was modified to make the channel wall hydrophobic. The Laplace valve utilizes the Laplace pressure derived from the surface tension, given by the Young-Laplace equation:

(1) $$P_{\mathrm{LP}\;}=-\frac{4\gamma \cos\theta }{D_{h}}$$

where γ is the surface tension, θ is the contact angle, and *D*_h_ is a hydraulic diameter, given by *D*_h_ = 4*A*/*P* (*A*: cross-sectional area, *P*: wetted perimeter). First, water was injected into the plug launcher at a pressure lower than the Laplace pressure (*P*_IN_ < *P*_LP_). Then, air was injected into the channel to form the micro liquid plug (*P*_IN_ < *P*_LP_). Finally, a pressure higher than the Laplace pressure was applied to drive the liquid plug in the acceleration channel (*P*_IN_ > *P*_LP_).

Table 1 lists the parameters of the seven microchannels used to create plugs with various diameters and lengths (0.4–58 nL in volume) in the experiments. These microchannels were fabricated from a glass substrate using two-step photolithographic wet etching [19]. The cross-sectional shape of the channel was rounded rectangular. The wetted perimeter of the channel was approximately estimated using the following equation: *P* ≈ 2*W* − 2*D* + π *D*, where *W* is the channel width and *D* is the channel depth. From this equation, the wetted perimeter was 174 μm for channels 1–5, 348 μm for channel 6, and 503 μm for channel 7. The width and depth of the Laplace valve were 40 μm and 10 μm, respectively. The hydrophobic channel wall was prepared using an amorphous fluoropolymer (INT-332VE, NI material). The static contact angle was θ = 113 ± 0.5°, as measured by a contact angle meter. Therefore, the Laplace pressure was calculated to be *P*_LP_ = 7.2 kPa using a surface tension of γ = 72.3 mN/m at the water-air interface.

A single liquid plug moving in the gas phase through a microchannel driven by an air pressure was observed. The plug position in the captured images was determined with a spatial resolution given by pixel size/magnification, and the plug velocity was calculated as the displacement of the plug front/time.

## 3. Results and Discussion

### 3.1. Behavior of Micro Liquid Plug in Microchannel

**Figure 2:**
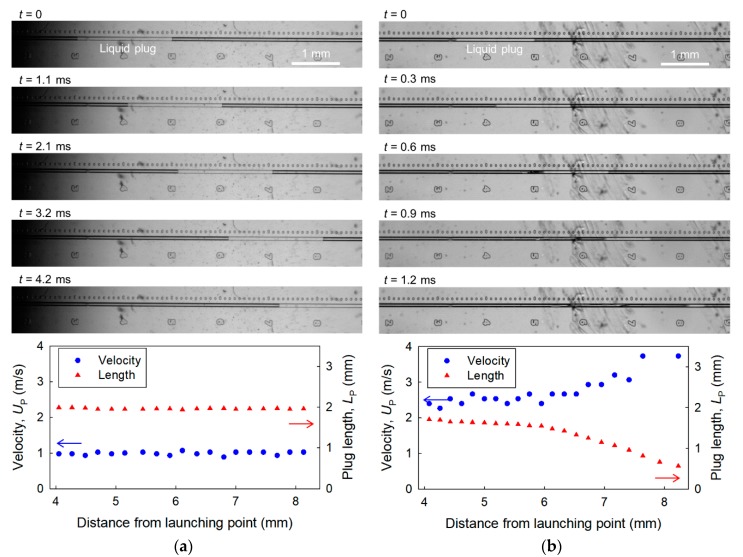
Images of a micro liquid plug moving through a microchannel with *L* = 2.00 mm and *D*_h_ = 39 μm, and velocity *U*_P_ and length of liquid plug *L*_P_ as functions of the distance between the launching point and the plug front at applied pressures of (**a**) 100 kPa and (**b**) 1600 kPa. *t* = 0 is the time when the plug front was at a distance of 5 mm from the launching point.

Figure 2 shows the motion of an accelerated liquid plug in the microchannel with *L* = 2.00 mm and *D*_h_ = 39 μm. At an applied pressure of *P* = 100 kPa (Figure 2a), the liquid plug moved in the microchannel at a constant velocity of *U*_P_ = 0.99 m/s, maintaining its initial length of *L*_P_ = 1.96 mm. In contrast, at an applied pressure of 1600 kPa (Figure 2b), the liquid plug started to split from its tail when the plug front was at a distance of 4.4 mm from the launching point, and the plug velocity increased with decreasing length of the main liquid plug. The rapid splitting of the liquid plug into large fragments occurred likely due to Rayleigh instability. Then the liquid plug transitioned into annular flow, and part of the annular flow often transitioned into satellite plugs because of the fusion of the liquid layer on the wall (*t* ≥ 0.6 ms in Figure 2b). The main liquid plug finally disappeared, which is consistent with our previous study [16].

**Figure 3:**
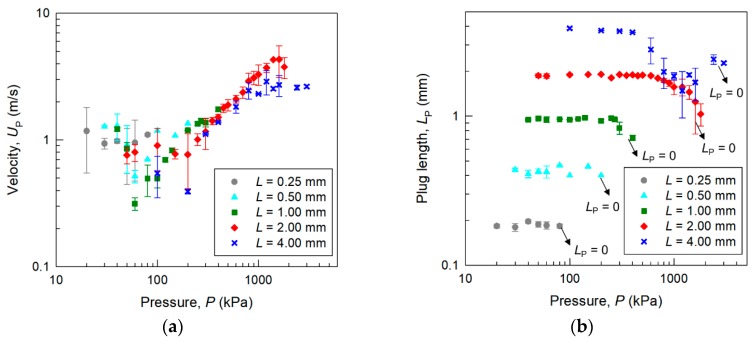
(**a**) Velocity *U*_P_ and (**b**) length of plug *L*_P_ at a distance of 8 mm from the launching point in a microchannel with *D*_h_ = 39 μm and various lengths of the plug launcher, *L* = 0.25–4.00 mm, as functions of applied pressure.

Figure 3 shows the velocity *U*_P_ and length *L*_P_ of the main liquid plug in microchannels with *L* = 0.25–4.00 mm and *D*_h_ = 39 μm as functions of the applied pressure at a distance of 8 mm from the launching point. Each data point is the average of 3–4 measurements, and the error bar indicates the standard deviation. Data obtained in our previous study [16] are also included. Interestingly, the plug velocities in the channels are approximately 1 m/s for 10–100 kPa, and then increase with further increases in pressure. The plug length starts to decrease from its initial length at a certain pressure. The velocity of shorter plugs (*L* ≤ 0.5 mm) has larger fluctuation than that of longer plugs, probably because longer plugs are more stable, having a broad wetting area on the channel wall. However, just before the splitting of the liquid plug, even longer plugs become unstable, and in this state both the velocity and length of the plug have large fluctuation. These results suggest that the micro liquid plug with *L* = 2.00 mm can achieve the highest velocity when the channel size is *D*_h_ = 39 μm. When the plug length was *L* = 4.00 mm, the plug became unstable and the maximum velocity was lower than that of the liquid plug with *L* = 2.00 mm. This result suggests that there may be an optimal balance between fluid resistance and plug stability. The maximum velocity of a liquid plug without splitting was 1.5 m/s, and that with splitting was 4.3 m/s.

**Figure 4:**
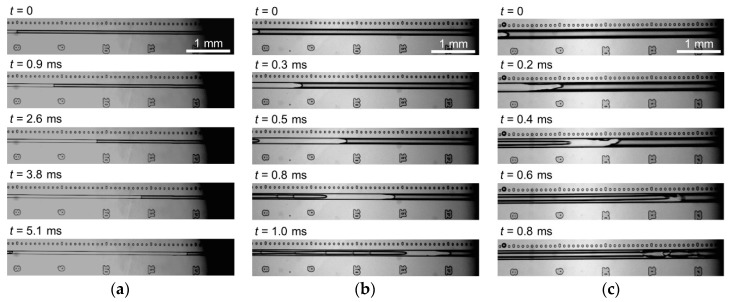
Images of micro liquid plug moving through microchannels with (**a**) *D*_h_ = 39 μm, (**b**) *D*_h_ = 79 μm, and (**c**) *D*_h_ = 116 μm and *L* = 4.00 mm at an applied pressure of 400 kPa. *t* = 0 is the time when the plug front was at a distance of 8 mm from the launching point.

Figure 4 shows the motion of an accelerated liquid plug in microchannels with *L* = 4.00 mm and *D*_h_ = 39–116 μm at an applied pressure of *P* = 400 kPa. Videos of the liquid plug moving through the channel are presented in the Supplementary Material (Videos S1–S3). Since the fluid velocity is proportional to *D*_h_^2^ in laminar flows, a higher velocity of the liquid plug in the larger channel was expected. However, the liquid plug easily split in the larger channel because of the reduced effect of surface tension, which is proportional to *D*_h_^−1^. As shown in Figure 4b,c, the transition from liquid plug flow to annular flow was more obvious for channels with *D*_h_ values of 79 μm and 116 μm than that for the smaller channel with *D*_h_ = 39 μm (Figure 2b). The liquid plug in these larger channels was broken up even at an applied pressure of about 100 kPa. Consequently, even when the channel size was increased to *D*_h_ = 79 μm or 116 μm, the maximum velocity of the liquid plug without splitting was 1.1 m/s and that with splitting was 5.2 m/s, which are comparable to the results for a channel size of *D*_h_ = 39 μm.

### 3.2. Velocity of Accelerated Micro Liquid Plug

**Figure 5:**
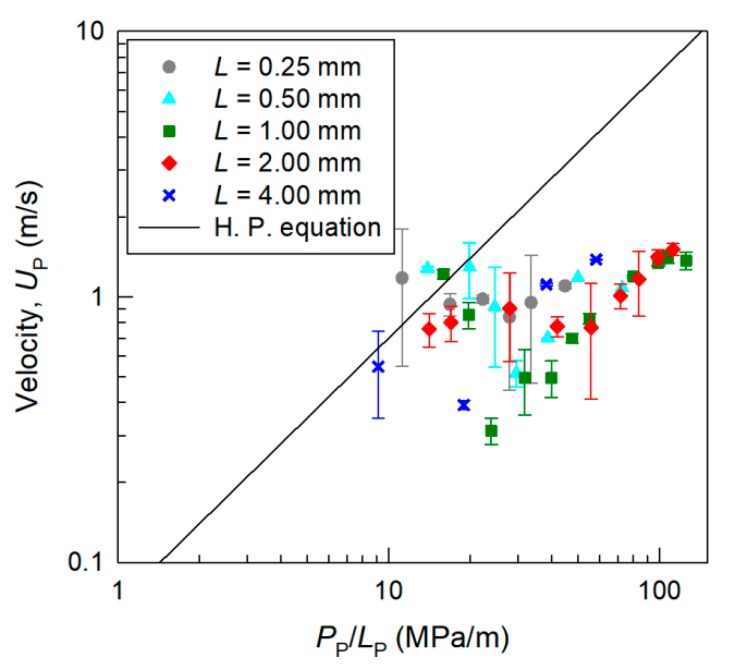
Acceleration efficiency of a micro liquid plug in microchannels with *D*_h_ = 39 μm and various lengths of the plug launcher *L* = 0.25–4.00 mm. Relationship between the pressure gradient in the plug (*P*_P_/*L*_P_) and plug velocity *U*_P_.

From the results, we investigated the dominant factors of the velocity of a micro liquid plug in the gas phase in a microchannel. We estimated the pressure loss of the micro liquid plug, *P*_P_, considering the pressure losses in the tubes, inlet channels, plug launcher, Laplace valve, and acceleration channel, which were filled with air. As shown in Figure 5, the relation between the plug velocity *U*_P_ and the pressure gradient in a micro liquid plug *P*_P_/*L*_P_ was estimated and compared with the Hagen-Poiseuille equation assuming single-phase laminar flow (since Reynolds number was 10^2^), given by:

(2) $$U_{P}=\frac{D_{h}{}^{2}}{16\mu }\frac{P_{P}}{L_{P}}$$

Here, we discuss the velocity of the liquid plug before splitting in a channel with *D*_h_ = 39 μm because the plug motion is considered to be in the steady state, and no fragments of the liquid exist in the channel. When the pressure gradient in the plug is about *P*_D_/*L*_D_ = 10 MPa/m, the velocity of the liquid plug is similar to that calculated using the Hagen-Poiseuille equation. However, with increasing pressure gradient, the velocity of the liquid plug increases very slightly; the velocity at a pressure gradient of 100 MPa/m is about 20% of that calculated using the Hagen-Poiseuille equation.

Several factors may contribute to the nonlinear plug velocity as a function of the pressure gradient. First, the Laplace pressure at the gas-liquid interface in the direction opposite to the pressure drop caused by the concave shape of the plug tail was considered. From Equation (1), the maximum Laplace pressure in the channel with *D*_h_ = 39 mm is *P*_LP_ = 7.4 kPa (θ = 180°). The pressure drop at the liquid plug was 10^1^–10^2^ kPa, which is higher than the maximum *P*_LP_. Hence, the effect of the Laplace pressure on the velocity of the liquid plug is considered to be small, especially for large pressure gradients *P*_P_/*L*_P_.

Second, the influence of the volume of the gas dissolved in the liquid plug at high pressure, which can reduce the pressure applied to the plug, was considered. From Henry’s law and the ideal gas law, the volume of gas dissolved in a nL liquid plug was calculated to be 0.01–1 nL, which is negligibly small compared to the volume of the microchannel, sample reservoir, and compressed air tank in the pressure control system. Hence, the influence of the dissolved gas on the terminal velocity of the liquid plug is considered to be negligible.

Other factors such as the effects of circulating flows inside a segmented micro plug [5,20] and gas flow at the corners of a hydrophobic microchannel through the liquid plug were considered. In order to determine the dynamics of the liquid plug, further investigation using a more sophisticated experimental setup, such as one that utilizes high-speed confocal microscopy, is required. In addition, although several theoretical models of pressure drop in segmented micro liquid droplets have been proposed [21,22], these models assume a segmented gas-liquid flow in hydrophilic microchannels where a thin liquid film exists on the channel wall. Therefore, the dominant factors responsible for the nonlinear plug velocity as a function of the pressure gradient in a hydrophobic microchannel are still unclear and will be examined further in future work.

### 3.3. Split of Accelerated Micro Liquid Plug

The dominant factor responsible for the splitting of a liquid plug was investigated. Here, surface tension maintained the plug shape and a force exerted by the fluid split the plug. Two nondimensional numbers characterize the liquid droplet and plug flows, namely the capillary number μ*U*_P_/γ (viscous force/surface tension) and the Weber number *We* = ρ*U*_P_^2^*D*_h_/γ (inertial force/surface tension), where ρ is the density of the liquid (998 kg/m^3^ for water). In the present study, since the Reynolds number is *Re* = *We*/*Ca* = 10^2^, the inertial force has a more dominant effect than that of the viscous force on the liquid droplet flow, as described in a previous study [7]. Therefore, here we considered *We*.

**Figure 6:**
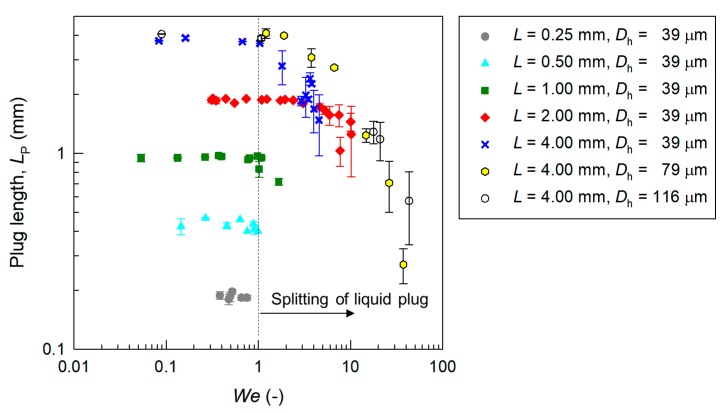
Relationship between the Weber number of the liquid plug *We* and the plug length *L*_P_ in microchannels.

Figure 6 shows the relationship between the Weber number *We* and the plug length *L*_P_. It is clear that the accelerated liquid plug in the gas phase for all channel sizes splits and disappears at *We* > 1. Qualitatively, when the air pressure was applied, the liquid plug was driven and an inertial force was exerted. When the inertial force was larger than the surface tension maintaining the plug shape, the breakup of the liquid plug occurred, as shown in Figure 2b and Figure 4b,c. The results suggest that the threshold is *We* ≈ 1. Therefore, we found the critical factor responsible for the splitting of a micro liquid plug in the gas phase in the hydrophobic microchannel. When the Weber number is lower than 10^0^, the accelerated liquid plug maintains its shape, and the liquid plug can be transported stably without splitting. When the Weber number is higher than 10^0^, plug splitting occurs. These results can be used for the design of microfluidic devices that utilize liquid droplets and plugs in the gas phase.

## 4. Conclusions

This study investigated the acceleration and splitting of a nL micro liquid plug in the gas phase moving through a hydrophobic microchannel by observing the plug motion using a high-speed CMOS camera. Experiments were conducted using 0.4-nL to 58-nL liquid plugs with precise size control in microchannels with hydraulic diameters of 39–116 μm. The liquid plug with a smaller diameter was more stable than that with a larger diameter due to surface tension. In the present system, the maximum velocity of the liquid plug without splitting was 1.5 m/s, and that with splitting was 5.2 m/s. The velocity of a single liquid plug was approximately 20% of that calculated using the Hagen-Poiseuille equation assuming single-phase fluid flow when a pressure gradient higher than 10 MPa/m was applied to the liquid plug. In addition, it was found that an accelerated liquid plug in the microchannel splits when the Weber number (a ratio of the inertial force to the surface tension) becomes higher than 10^0^. These results can be used as a design guideline for achieving the stable transport of a micro liquid plug in the gas phase in a microchannel at the highest velocity without splitting for chemical processing at high efficiency and high throughput. Since the experiments were conducted in well-controlled conditions, the results will contribute to the development of various microfluidic applications using segmented liquid droplets and plugs in the gas phase [7,13,14,23,24].

## Figures and Tables

**Table 1:** Parameters of microchannels used in the experiments.

Microchannel	Launcher Length, *L*	Width, *W*	Depth, *D*	Hydraulic Diameter, *D*_h_
Channel 1	0.25 mm	70 μm	30 μm	39 μm
Channel 2	0.50 mm	70 μm	30 μm	39 μm
Channel 3	1.00 mm	70 μm	30 μm	39 μm
Channel 4	2.00 mm	70 μm	30 μm	39 μm
Channel 5	4.00 mm	70 μm	30 μm	39 μm
Channel 6	4.00 mm	140 μm	60 μm	79 μm
Channel 7	4.00 mm	200 μm	90 μm	116 μm

## Supplementary Materials

The following are available online at http://www.mdpi.com/2072-666X/9/9/423/s1, Video S1: Accelerated liquid plug in microchannel with *D*_h_ = 39 mm and *L* = 4.00 mm at an applied pressure of 400 kPa (1/2000 speed). Video S2: Accelerated liquid plug in microchannel with *D*_h_ = 79 mm and *L* = 4.00 mm at an applied pressure of 400 kPa (1/2000 speed). Video S3: Accelerated liquid plug in microchannel with *D*_h_ = 116 mm and *L* = 4.00 mm at an applied pressure of 400 kPa (1/2000 speed).
